# One size doesn’t fit all: a thematic analysis of interviews with people who have stopped participating in Narcotics Anonymous in Norway

**DOI:** 10.1186/s13722-020-00191-w

**Published:** 2020-05-24

**Authors:** John-Kåre Vederhus, Magnhild Høie, Bente Birkeland

**Affiliations:** 1 Addiction Unit, Sørlandet Hospital HF, Po. Box 416, 4604 Kristiansand, Norway; 2 University of Agder, Grimstad, Norway

**Keywords:** Substance use disorders, Self-help groups, Narcotics Anonymous, Norway

## Abstract

**Background:**

For persons with substance use disorders (SUDs), 12-step groups (TSGs) are the most available and used peer-based recovery resource, worldwide. However, disengagement is common, and attrition may partly be due to practices and procedures within these groups that are unacceptable to a portion of the population with SUDs. Our overall aim was to identify problematic issues related to Narcotics Anonymous (NA) participation in Norway, to inform addiction professionals’ strategies when referring persons to addiction-related self-help groups (SHGs).

**Methods:**

In this qualitative study, we interviewed ten individuals who had previously participated regularly in NA for at least 6 months, to examine their reasons for disengagement. We interpreted the interviews using thematic analysis.

**Results:**

We identified three themes: (1) **‘**The model did not fit’, either the strategies utilized in NA (e.g., meeting format and step working) or NA’s explanatory model of addiction, (2) ‘Negative experiences spurred frustration’, and (3) ‘The safe place can become a cage’. The respondents believed that a main aim of recovery was reintegration into society, such that SHG participation should not be an end goal, but rather a platform for normalization back into society. Despite their negative experiences and strong critique, respondents still regarded NA as a valuable recovery resource, but pointed out that one size does not fit all.

**Conclusion:**

Addiction professionals should recognize possible problems related to TSG participation, to help prevent negative experiences and possible harms to individuals. Professionals should also inform individuals about alternative support groups, to help them find the recovery resource best suited to them.

## Background

Referral to addiction-related self-help groups (SHGs) is considered a standard component of the treatment regimen for patients with a substance use disorder (SUD) [1]. Such groups typically encourage finding positive role models, achieving abstinence-oriented norms, and bolstering members’ self-efficacy and coping skills [2]. Treatment guidelines specifically recommend the most available groups: the 12-step groups (TSGs), such as Alcoholics Anonymous (AA) and Narcotics Anonymous (NA). For example, the American Psychiatric Association’s treatment guidelines for SUDs state that referral to a TSG may be beneficial during all stages of the treatment process [3]. As the intensity of treatment services is decreasing, researchers have noted that SUD treatment has the proximal goal of fostering stable engagement in TSGs to provide clients with a support network following the discontinuation of formal treatment services [4–6].

However, high proportions of newcomers in TSGs soon disengage. For example, an analysis of AA member surveys revealed a 50% drop-out rate after just 3 months of starting [7]. While some participants may drop out due to poor motivation [4], it is also possible that attrition is partly prompted by TSG practices and procedures that are unacceptable to a portion of the population with SUDs [8]. Some individuals are dissatisfied with various tenets of 12-step programs, and seek out other SHGs with alternative change strategies. Notably, the founder of Women for Sobriety (WFS), Jean Kirkpatrick, perceived the 12-step program as being negative for women, particularly due to its focus on dependence on a Higher Power and on admitting “powerlessness”—i.e., that you should acknowledge your own inability to control your drinking/substance use and that you must let outside forces help you. Jean Kirkpatrick argued that to reduce guilt, women need a program that reinforces positive thinking about a woman’s abilities, thus instilling independence [9]. Accordingly, WFS uses a 13-step ‘New Life’ program that focuses on positive thinking and on believing in one’s own competency to overcome a lack of self-confidence and low self-esteem [10]—thus emphasizing a “strengths” perspective [11]. Recent empirical evidence from the Peer ALternatives for Addiction (PAL) study shows that compared to TSG members, members of alternative mutual help groups (e.g., WFS and Smart Recovery) have equivalent activity involvement and, unexpectedly, higher levels of cohesion and satisfaction with their group [12]. Although such findings do not directly demonstrate the efficacy of these alternative groups, the high satisfaction and cohesion are relevant to this subject.

Factors like those highlighted by Jean Kirkpatrick have been cited as arguments for identifying and encouraging alternatives to TSGs [8]. In the setting of present-day Norway, several alternative addiction-related SHGs exist, but TSGs still have a near monopoly in many regions [13]. Beyond the strict definition of SHGs [14], other resources are available—for example, addiction-related user organizations and non-governmental organizations (NGOs) are available to provide patients with a supportive environment through positive activities, such as leisure and social activities.

### The practices of NA: a 12-step fellowship

In the present study, we focus on NA—a fellowship that was formed in the 1950s by adapting the 12-step program of AA to other substances of use [15]. NA gained a foothold in Norway approximately 25 years ago, and its presence has been steadily growing. There are currently 134 weekly NA group meetings nationwide (~ 3 per 100,000 inhabitants)—which is close to the number of AA meetings. In larger cities there are daily meetings, while one weekly meeting is more common in rural areas.

As a background to understand attrition, here we describe the practices of this fellowship. The 12 steps of NA serve as a set of principles outlining a course of action for recovery from addiction. They can be considered as suggested change strategies, and consist of admitting that you have a problem (step 1), seeking help (steps 2, 3, and 11), conducting a “moral inventory” (steps 4–7 and 10), and making amends to those you have harmed (steps 8 and 9). Participants are also encouraged to help each other by sharing their story (step 12) [16].

The primary focus of NA is to provide a recovery environment in which participants can share their recovery experiences with each other, and the therapeutic value of one participant helping another is a basic principle of the program [17]. The primary activity is the group meeting, which is led by members themselves. Individuals participate by taking turns sharing their experiences of how to recover from addiction. The meeting format has a strong narrative tradition, with no dialogue or “group therapy”. The only prerequisite for participation is a desire to stop using mind-altering substances, and the goal of NA is to promote abstinence (i.e., being “clean”) from all such substances, including marijuana and alcohol. However, one can participate even if one is not completely abstinent, and participants are welcomed back at any time after a relapse; thus, the threshold for participation is low.

The groups also incorporate a mentor function, called “sponsorship”, whereby newer members can ask a more experienced group member to provide guidance outside of the group meetings. It is recommended that a member partner with a sponsor to do “step work”, i.e., successively work through the 12 steps to develop a deeper understanding of the steps and their relevance to one’s own life. As a part of this process, it is also recommended that the member acknowledge the nature of his/her own wrongdoings, expressed as “defects of character”, such as self-centeredness or resentment—i.e., to look beyond the mere actions. The rationale is that one must completely change the “old ways of thinking” to avoid starting substance use again ([18], p. 21). In step 4, a review is undertaken privately. In step 5, this review is turned into a social interaction when it is conveyed to a trusted person—often a sponsor.

NA has also adopted the organizational principles of AA: the 12 traditions (Table 1) that describe how to run the local groups [16]. These principles describe the fellowship as a “bottom-up” organization, in which each local group is autonomous, self-supporting, and self-governing (traditions 4, 7, and 9). There is an existing service structure, including group-elected service boards and committees, but the power is defined as lying at the base rather than in the structure [19]. NA texts emphasize the equal status of members, and a number of principles contraindicate the build-up of hierarchy. No one acts as a professional in the groups, and rotation of representation and leadership in the service structure is mandated. When decisions are made within a group, tradition 2 refers to the “group conscience”, which has come to mean decision making via discussions and hopefully consensus [19]. Several traditions (e.g., traditions 11 and 12) mention the external anonymity principle, which is a crucial element in maintaining equality and democracy. Although old-timers and experienced members are looked up to and often play a moral leadership role, the formal equality of members carries a more substantive weight than in many other organizations [19]. For example, tradition 12 states that principles should trump personalities, tradition 2 states that leaders should not govern but should rather be trusted servants, and a newcomer (evidently a person with the least seniority) is considered the most important in a meeting [20].

**Table 1:** The 12 traditions of Narcotics Anonymous [16]

1. Our common welfare should come first; personal recovery depends on NA unity
2. For our group purpose there is but one ultimate authority a—loving God as He may express Himself in our group conscience. Our leaders are but trusted servants, they do not govern
3. The only requirement for membership is a desire to stop using
4. Each group should be autonomous except in matters affecting other groups or NA as a whole
5. Each group has but one primary purpose—to carry the message to the addict who still suffers
6. An NA group ought never endorse, finance, or lend the NA name to any related facility or outside enterprise, lest problems of money, property or prestige divert us from our primary purpose
7. Every NA group ought to be fully self-supporting, declining outside contributions
8. Narcotics Anonymous should remain forever nonprofessional, but our service centers may employ special workers
9. NA, as such, ought never be organized, but we may create service boards or committees directly responsible to those they serve
10. Narcotics Anonymous has no opinion on outside issues; hence the NA name ought never be drawn into public controversy
11. Our public relations policy is based on attraction rather than promotion; we need always maintain personal anonymity at the level of press, radio, and films
12. Anonymity is the spiritual foundation of all our traditions, ever reminding us to place principles before personalities

### Objectives

Although attrition from TSGs is common, disengagement studies are scarce [21]. Moreover, there are few studies at all about TSGs outside the U.S. [22]. In the present study, we assessed participants who had been engaged in NA at some point in their life and had left for some reason. We wanted to learn of their experiences and examine why they disengaged. Their reasons for disengaging served as a proxy for identifying problematic issues related to TSG participation. To obtain a complementary view of their relationship with NA, we also explored what role NA had played in their own recovery. Finally, we examined whether they had advice that they would give to potential participants based on their own experiences. Our findings could potentially inform addiction professionals’ strategies when they refer patients to addiction-related SHGs in general, and particularly to TSGs.

## Methods

### Present setting and participants

In this qualitative study, we explored the views and experiences of respondents who had previously been regular NA participants for at least half a year but who had disengaged from the fellowship. An additional inclusion criterion was being in a stable recovery, which was left up to the participants to define—although as a timeframe, we indicated that the respondents should have been in recovery for preferably at least 2 years. We wanted to include participants who still defined themselves as in recovery after leaving, to avoid depreciation of their experiences and opinions. Respondents were recruited via NA attendees known by the authors, who contacted potential participants who had left the fellowship and asked if they were willing to participate. Additionally, user organizations and NGOs in the addiction field were asked if they knew potential participants, and a few respondents suggested other individuals who they thought would be interested. Willing participants were contacted by the researchers.

The respondents came from three large Norwegian cities, and included six men and four women aged 40–56 years (average age, 50 years, Table 2). Their duration of regular attendance in various NA home groups ranged from 0.5 to 11 years, with 8 of the 10 participants having attended > 90 meetings in their lifetime (two had attended > 500 meetings). Table 2 shows additional details about the participants, for example, how many years they had experienced problematic substance use.

**Table 2:** Participants’ demographic data and relevant substance use and NA-related information (N = 10)

Pseudonym	Age	Gender	Years of problematic substance use before NA	Years of regular NA attendance	Years in recovery after NA	Participations in support groups after NA?
Per	46	Male	28	0.5	2.5	User organization/other addiction-related support group
Kari	56	Female	24	0.5	20	AA/NGO
Hans	51	Male	27	2	9	None
Ann	52	Female	10	4	18	Other support group (not related to addiction)
Håkon	54	Male	20	3	2.5	User organization/other addiction-related support group
Celine	43	Female	7	4	12	None
Inge	40	Male	7	3	10	User organization/other addiction-related support group
Oda	53	Female	30	6	5	Other addiction-related support group/NGO
John	56	Male	32	7	3	Other addiction-related support group/NGO
Arne	53	Male	20	11	7	Occasional NA/AA attendance

*AA* Alcoholics Anonymous, *NA* Narcotics Anonymous, *NGO* non-governmental organization (non-profit)

### Data collection

To obtain a varied sample sufficient for elucidating the study objectives, we aimed to include at least 10 respondents. We further sought to include participants of both genders and who had different types and levels of support after leaving NA, to ensure the exploration of a variety of experiences. The first wave of interviews was conducted in 2015, and the second in 2017. Interviews lasted approximately 90 min, and were conducted by JKV and MH, or JKV alone. Seven respondents participated in individual interviews. The eighth respondent unexpectedly brought to the interview two friends who had also left NA. Since that interview was held at a faraway location with a limited timeframe for the return journey, we chose to conduct a group interview out of respect to those attending.

A thematic interview guide was developed. First, participants were asked to briefly narrate their life before joining NA, and how they became involved. Then respondents were asked to elaborate on their experiences in NA, how and why they eventually left, and whether they had subsequently obtained other sources of support. The interviews were conducted in Norwegian, digitally recorded and later transcribed verbatim. The participants’ quotations that are presented below were translated to English by a fluent English speaker.

### Analysis

The interviews were analyzed using inductive thematic analysis of data meaning and content across cases, with the aim of extracting and thematizing participants’ experiences with NA and their ways of leaving the NA fellowship [23]. The analysis entailed familiarization with the data, identification of important features relevant to answering the research question (coding), generating and reviewing themes, and choosing an informative name for each theme. When organizing the data, we clustered the sub-themes based on their content (codes), and identified preliminary themes. After discussion and re-organization, we arrived at the final themes. Illustrative quotations were identified, and assigned with pseudonyms. Finally, the analytic narrative was contextualized and discussed in relation to existing literature.

### Preconception

Two of the authors were familiar with TSGs and the 12 steps due to their clinical experience with SUD treatment. They had previously been involved in a Twelve Step Facilitation study, and had fundamentally positive preconceptions about TSGs [5]. The other author had less experience with the theme, but had previously undertaken a study about support groups for family members of persons with a SUD [24]. Such familiarity with the field under investigation is considered a resource, as it can provide a head start towards knowledge on the topic. However, as we here studied persons who had left NA, our preconceptions could also be considered a potential obstacle for trust building. To positively affect the respondents’ willingness to share information, we clearly stated our research goals to participants before the interview, and emphasized that we also wanted critical information about their participation.

## Results

With regards to the time-frame in the fellowship and degree of affiliation with NA, the participants described different courses. Per and Kari were early drop-outs; they did not feel at home and did not become deeply involved (Table 2). Hans, Håkon, Ann, and Celine were quite involved for 2–4 years but gradually grew away from the fellowship, partly because they established their lives anew with studies, work, family, and other friends. As they did not need NA as much, their exit appeared to represent a “natural course” of disengagement.

*Maybe it was because … you know … I steadily became a more productive member of society… right, in parallel with my NA participation. Perhaps that bit about being a regular member of the community grew stronger… as time went by… and it was like… eh, what do I really want? And… who am I? Who do I identify with? Eventually, maybe the identification became stronger with those … I had more in common with and did more things together with “ordinary” people. (Ann)*

Inge had a similar period in NA (3 years), and described himself as a zealous adherent. However, after relapsing to heavy opioid use, he found it increasingly difficult to return to the fellowship; he became disillusioned and lost faith in the “NA way”. While in NA, he had developed a very negative attitude towards opioid maintenance treatment (OMT); however, he felt that to stay alive he had to give in and apply to an OMT program. Oda and John stayed in NA even longer (6–7 years), but their frustrations with the fellowship gradually grew. They felt that their needs were not met and wanted something “more than” or “different from” NA. Eventually, they found a supportive NGO that was more suited to their needs, and became involved there instead. Arne was an “old-timer” and was heavily involved in NA for over 10 years. However, he eventually felt that participation reduced his quality of life rather than enhancing it, and thus disengagement from the fellowship became a necessary liberation process for him.

### Problematic issues related to TSG participation

Some of the reasons for disengaging are implicitly mentioned in the above-described patterns of disengagement. Below, we further elaborate on the underlying emotional and individual reasons for disengagement. Table 3 presents the thematic analysis (including codes, preliminary themes, and final themes) related to this main study objective.

**Table 3:** Overview of the analytic process of the theme “Problematic issues related to TSG participation”, including codes, preliminary themes, and final themes

Codes	Preliminary themes	Final themes
Difficulty with strategy, e.g., meeting format, sharing, step working Double trouble (e.g., trauma and psychiatric co-morbidity) and the need for something more than or different from NA Perceived negative focus (e.g., powerlessness, defects of character, demands of honesty to show willingness) Everything is hinged on addiction NA presented as the only solution to addiction	Strategy doesn’t fit Explanatory model doesn’t fit	The model doesn’t fit
Negative experiences with sponsor Anonymity breaches Conformity pressure Criticism is not welcomed Seasoned members not living as they “teach” Not able to live up to success criteria Relapse and negative emotions	Negative experiences in the social environment Perception of being a second-rate member	Negative experiences spurred frustration
Natural course of disengagement Continued participation has no additional benefits Participation limits quality of life Ambivalence about breaking out	Life ought to be more than NA Perceptions of being “stuck”	The safe place can become a cage

#### The model did not fit

The respondents’ objections to the NA model of recovery took various forms. Some had negative feelings about some of the strategies utilized in NA. For example, Per and John were not comfortable with the narrative tradition in group meetings because both felt too “messed up” due to their psychiatric comorbidity and had difficulty sharing. For Per, these thoughts were deepened when he experienced a more dialectic form in an SHG through a user organization. He felt that the dialogue and feedback in their group meetings were more beneficial for him, making him more comfortable, such that he settled down there instead. After a few years, he was even established as a leader in the SHG. Similarly, John found an NGO that ran an educational program on how to cope in recovery, and found their focus on social and leisure activities (e.g., football) to be more attractive than the typical group meeting format in NA. Both John and Per were also critical of other parts of the NA approach, e.g., they resisted the step-working practices and felt that the expectation of involvement in a strict step-working structure was a burden. After Per left NA, he eventually looked seriously at the 12 steps and found them to be sensible, but realized that he had intuitively utilized similar strategies with the help of the alternative SHG that he had settled down in.

Some participants also expressed objections to the explanatory model of the 12 steps. Celine said *“they hinge everything on addiction”*, indicating that she felt that alternative models were more relevant for her. She had a serious trauma experience in her late teens, when she was given a rape drug and was raped by two men. During step 5, she recollected the incident and her sponsor’s response was “look what our addiction has led us into”. At the time, she was surprised by her sponsor’s response and intuitively objected to it, but she was unable to express her objections or even formulate to herself why she strongly reacted to the sponsor’s feedback. She no longer approached her sponsor after that incident, but rather only utilized other resources available in the group, e.g., the meetings and the literature. Retrospectively, she was quite clear that she had acquired an addiction and needed to take accountability for it. However, in contrast to her sponsor’s view, she realized that it was not the addiction that led her into the traumatic experience—it was the other way around.

“*After that [being raped], my drug use went wild. It was the most sensible thing I could have done at that time. Otherwise, I would have been locked up in a psychiatric ward or committed suicide.” (Celine)*

Several respondents criticized a perceived negative focus in the 12 steps and step-working practices—for example, the focus on the powerlessness principle in step 1, and on “defects of character” in step 6. Participants perceived that the powerlessness principle conveyed an attitude of being unable to accomplish anything on your own, having a lesser value, or feeling devaluated.

*You are nothing in yourself; you cannot trust yourself when you are on your own. All knowledge about how to live your life … you don’t have it in you. By yourself, you are in bad company… [i.e., “an addict alone is in bad company”](Arne)*

Håkon even aligned the powerlessness principle with being a “loser”, and Celine stated that it went against her view of humanity. She had felt sorrow when she heard that “your best thoughts have led you here”, and she stated “I felt the underlying truth was: I am worth nothing”. Similarly, Kari found herself questioning whether she ever did anything right, implying that the program undermined independence and self-confidence:

*It was like … it was not right to be happy. You should be humble … humiliated … humble and grateful. It was like … a strange mixture of … you should remind yourself of your own badness all the time and, nonetheless, be humble and grateful. If you tried to be kind to those you had been with, it was deemed as codependence. Whatever you did, it was pathologized and dragged down. I had this feeling: am I never doing anything right? (Kari)*

An overall view was that many of the respondents desired a higher focus on a “strength perspective”.

#### Negative experiences spurred frustration

As illustrated by Celine’s experience in step 5, the relationship with one’s sponsor could be critical and spur negative emotions.

*It is very vulnerable. For some, doing the fifth step with a sponsor can be liberating. For me it wasn’t. It did not give me the freedom I hoped for. I sat there and described my traumatic experiences to another person. I remember that I felt she didn’t have a clue as to what I was talking about. (Celine)*

Kari had a similar experience with her sponsor. As a child, she had been molested by an elder in her church. During step 5, she felt that she was misguided on how to cope rather than receiving sound guidance. Her sponsor told her that she ought to forgive her molester. When she later talked about it with a priest, he simply asked: “Has he [the molester] asked for forgiveness?”—indicating that forgiveness was not a primary concern when the molester denied his misdeeds. Her experience in step 5 left her with frustrated feelings regarding forgiveness, and locked her to the molester and the traumatic events, rather than facilitating a healing process. She also pointed out that her sponsor did not make a connection from her childhood experience and her later experiences of being sexually assaulted when she was intoxicated. In the latter, she felt that she might have been considered to be more “responsible” for the risky situation she had put herself in. In summary, Kari’s experience with the 5th step solidified her feelings of guilt rather than liberating her. Due to her background, she was also uncomfortable with the friendly “hugging” among members before each meeting. As this became too much for her, she instead began attending AA meetings, where this practice was less prevalent.

Some respondents had experienced breaches of the anonymity principle, learning that vulnerable stories shared in meetings were discussed among other members and rumors were spread about them. Håkon addressed the person who originated a rumor he learned was spread, and confronted him in an attempt to stop the spreading. He interpreted the rumor spreading and slander as being due to jealousy or as a means of raising one’s own status at the expense of others.

Several other negative experiences related to the social environment were described. Despite the non-hierarchical ideal, the respondents saw a clear hierarchical structure with different layers of membership. The hierarchy depended on a person’s standing in the fellowship, and was based on certain “success” criteria—first and foremost, the length of time you had been abstinent. Per stated that: “*The counting of days seems to be the most important thing in the world for them”.* Other hierarchal criteria included whether you thoroughly worked the steps (and made it known to other members), shared in meetings, or had many sponsees. The hierarchy was supported by expressions typically used in the fellowship, such as “stick with the winners”. If you were unable to live up to the standards on these outwardly visible areas, there was inevitably a risk of feeling inferior. Kari simply stated: *“You are looked down on if you are not working the steps”*, leading to a self-perception of being a second-rate member.

Nothing accentuated a feeling of inferiority more than a relapse. Celine, Arne, and Per perceived an “all or nothing” attitude in the fellowship. When a person returned to the group after a relapse, rather giving them a “clap on the shoulder” and meeting her/him with a supporting attitude, they rather felt that the group met the person with skepticism and excessive focus on the defeat.

*When you have had a relapse, it’s because you have not worked the steps well enough … or you were deceptive when you relapsed… you planned it. We say yes, but in fact we disagree with what we are told … and that also becomes wrong, because they have succeeded so well, right? (Kari*)

The long-timer, Arne, had a relapse himself after 10 years. He spoke to his sponsor about it, but he felt that some of the other old-timers indicated that this was not enough. They seemed to think that he should preferably have made a plenary “confession”, probably because they thought both he and the fellowship should learn from the incident. When you have had a relapse, you start counting drug-free days from zero again, and there is inevitably a risk that you feel that you are “back to the start”. He felt that other seasoned members had little regard for what he had achieved during his years in recovery, and seemed to place greater emphasis on his failure. Per described a similar experience:

*I was very proud when I had 30 drug*-*free days, but then I had a lapse on alcohol. I bought two six*-*packs [of beer], but after the first six*-*pack, I realized that I didn’t want to continue and I stopped. When I came clean to the NA meeting on Monday, I was very satisfied with myself because I had stopped and was determined to continue attending meetings. But I wanted to be honest and told about it in the group. Instead of telling me how good it was that I had stopped, they focused on “What a pity, now you have to start on zero again”. (Per)*

In line with the descriptions about the invisible hierarchy, participants also described conformity pressure, in that you ought not question or be critical of acknowledged truths. Hans described himself as an independent person who liked to do things his “own way”. He took pride in having independent thoughts, and simply could not immediately accept everything told to him. He felt that the conformity pressure was related to fears of those who had status in the fellowship, with questions and critiques arousing fear and opposition from those who were in a position to defend the truths. In one serious example, Hans had been openly critiqued by a seasoned member during a meeting, and had felt very humiliated. He acknowledged that the critique was likely prompted by his own tendency to be provocative. Hans was aware that he had used sharing during the group meetings to provoke some of the more seasoned members, and they might have felt that their authority was threatened. After being ridiculed in the meeting, he later realized that it might partly have been brought about by his mentioning that he was about to start a university education, and he learned that the seasoned member had once had the same dream but had not dared to pursue it. Hans eventually came to a point where it was no longer constructive for him to stay in NA—neither for himself nor the fellowship—and the only reasonable solution was to leave. Håkon had similar frustrated feelings. He became so fed up with the negative social experiences in NA that he even said that if he had not left, he would have started to use drugs again due to his discouraging experiences in the fellowship.

Celine’s initial glorified feelings about the fellowship faded away over time, and she begun to question the moral leadership of the persons she had previously admired and looked up to. She perceived that some of the seasoned members hid behind a perfect façade based on their status of longtime abstinence, but the rest of their lives might have been “a mess”. She remembered thinking “So much for honesty and willingness”. Similarly, Hans thought “Anyone can read and recite the literature, but you should rather live by it”. Celine wished that when she started to feel skeptical, she had talked to someone within the fellowship, who might had been able to frame her negative thoughts. Instead, her negative thoughts led her to view the fellowship with increasing skepticism, and she eventually drew away.

#### The safe place can become a cage

After surgery during his late recovery, Arne experienced re-activation of a capsulated trauma. As a teen, he had been raped by male inmates in a prison. Following his surgery, he had to obtain professional help to cope with post-traumatic stress. After an extensive therapeutic process, he felt more safe and secure, and his underlying fear that he would use drugs again diminished. He then felt that NA was too narrow a framework for his life. He realized that the trauma had greatly influenced his compulsive drug use, as well as his recovery process. He had tried to stay inside the box (i.e., do as was suggested: go to meetings, work the steps, talk with your sponsor), and he was afraid to be careless or negligent and end up in a “danger zone”. He recognized that he had been constantly anxious about whether he had done enough to secure his recovery. Ultimately, NA participation made him feel uncertain and fearful, and reduced his quality of life rather than enhancing it; thus, he felt he had to “break loose” from the fellowship to be able to feel relaxed. At the time of the interview, he still attended a few occasional meetings but he had distanced himself and exercised a “selective hearing”.

Several respondents made similar comments, expressing the point of view that the recovery fellowship ought to be a platform to get you back into society as an “ordinary citizen”, not an end goal in itself. If the platform becomes your whole life, it will eventually be a narrow-minded life; i.e., NA alone is not enough, and there is a risk of becoming “stuck” there. The participants described examples of members who had grown comfortable within the fellowship but who seemed unable to establish themselves anew in the “normal” society. Consequently, they seemed to have a poor quality of life outside the setting of NA. Such persons were mentioned by the respondents as examples of narratives that they did not want to find themselves in, and their disengagement from NA was one means of avoiding such a course. However, when respondents began to consider leaving, they commonly had ambivalent thoughts, like “Can I trust myself if I want to leave the fellowship?” and “Am I able to stand my own ground?”. It was a strong saying in the fellowship that when you leave, you will not manage on your own and you will certainly relapse. This made participants feel trapped in a “checkmate” position when they thought about leaving: “Damned if you leave and damned if you don’t”.

### What NA has meant and current thoughts about NA

Several respondents mentioned that they welcomed the opportunity to talk about their experiences in NA, with one respondent even expressing that it was therapeutic for him. Celine said that “when you have left NA, there are not many places or opportunities to talk about it …. You are reckoned almost like an apostate”. Despite their strong critiques and frustrated feelings, the respondents still respected NA as a recovery fellowship. Per described it almost in terms of a hate–love relationship, saying “I cannot hate the fellowship that saved my friends, but NA was just not for me”. Arne said it would be terrible if his critique of the fellowship should cause NA to disappear or no longer be recommended, and Kari praised those who helped others by “keeping the doors open”.

The respondents who were engaged in user organizations and NGOs said that when they visited treatment facilities to talk about and recommend their own organization, they also mentioned NA as a possible resource for potential attendees. However, they did not perceive that this recommendation was reciprocated; NA members only recommended NA when they visited the same facilities. To some degree, they interpreted this as an arrogance of NA participants, i.e., the attitude that only NA “works”, and they regretted that NA participants did not praise or recommend other recovery resources.

Many of the respondents also held the 12 steps in high regard, and still used them in their continued recovery. Kari stated that “There’s nothing wrong with the 12 steps; the problem is how they are presented and practiced”. Similarly, others made a distinction between the positive influence that the program had on them versus their negative social experiences in NA. Ann stated that she saw the 12 steps as a brilliant recipe for life in general, not only for coping with addiction. Many years after leaving NA, she encountered personal problems (a difficult divorce that caused a near mental breakdown), and she re-defined and renewed her relation to a Higher Power and consciously used the 12 steps to cope with her difficult life situation. Additionally, those who were engaged in a user organization saw that the courses they arranged were influenced by many of the ideas from the 12-step program, although they termed the themes differently:

*It’s about getting to know yourself: your values and your dreams. But we use other words for it, like empowerment and recovery … but it’s really about the same issues. (Inge)*

Almost all respondents—with the exception of the two who spent the shortest time in NA—praised NA for its importance in their own early recovery. When asked to consider where they likely would have been now without NA, even those who were most critical thought that they would probably have fared much worse. They acknowledged that without a supportive fellowship when they wanted to stop using drugs, they would have felt that they were in a “vacuum”. Even though Hans thought that seasoned members may have viewed him as a “thorn in the flesh” when he was in the fellowship, he said that he “would not have been clean now and would probably be dead without NA”. Celine praised the program and the fellowship as a vital resource during the beginning of her recovery, which helped her establish her life anew. Most importantly, she met a new drug-free network of friends in NA, some of whom remain close friends. She was also quite clear that some of the NA absolutes had been necessary in her early recovery—for example, although she now occasionally used some alcohol, she was glad that alcohol use was a non-issue during her early recovery due to her NA affiliation.

### Advice to potential participants

When asked about advice that they thought it would be wise to convey to other possible NA attendees, the respondents had several propositions. Based on their own experiences, Per, John and Oda were concerned about how persons with psychiatric comorbidity would fare in NA. They were quite certain that they would encounter problems and thought that alternatives, like the NGO or the user organization that they participated in, would be preferable.

Several respondents suggested warnings about the first honeymoon phase in NA, during which the member thinks she/he is in “heaven” and everything is fantastic. Looking back, they regretted having openly shared some things in meetings and they warned against “putting your whole soul on the table”. This was due to their skepticism regarding how well the anonymity vow worked, especially since many of the groups they had attended were held at relatively transparent locations. They advised that you should not “confess” in a meeting something that you would not want publicly known. They thought that some new attendees might not be sufficiently able to discern what should be shared individually with a sponsor or other trusted persons rather than in a meeting.

Although many of the respondents were proponents of individualistic thinking, they still advised against thinking entirely on your own too early in recovery. After being in recovery for 4 years, Ann wanted to occasionally use some alcohol, and did not think it would be a problem for her. She respected the honesty principle and the fellowship, and thus realized that she had to leave NA if she wanted to use alcohol. However, she would not suggest that others try using alcohol, at least in early recovery. Her general recommendation was to be careful and not to trust yourself when you had thoughts contrary to the recommendations of the fellowship. Even Hans, who was proud of being an independent thinker, admitted that he regretted having left the fellowship too early. He felt that he probably would have had an easier and faster personal growth in recovery if he had been a bit less in opposition and stayed longer.

An overall view, which was quite obvious to the respondents based on their own experiences, was that “one size doesn’t fit all”, and it would be ideal to have a menu of recovery fellowships available. If you try NA but don’t find it comfortable, you should also try other recovery resources.

## Discussion

### A differentiated menu of support groups is needed

Our findings that some respondents disliked NA are similar to recent findings of the large quantitative PAL study. In qualitative open-ended responses, PAL respondents who had left TSGs reported the things they disliked most about TSGs, which included the “concept of powerlessness”, “pressure to speak and do service work”, and “sponsorship” [25]. However, the findings of our qualitative study obviously cannot reveal the whole truth about NA. As the respondents themselves pointed out, NA was an important factor in their own recoveries, and they recognized NA as a potentially useful recovery resource for persons with addiction. Thus, their main message did not seem to be that NA should be deprecated. Rather, they highlighted the usefulness of a differentiated menu of support groups for persons recovering from addiction, and the need to also appreciate these other support options. Additionally, it is likely that a person may benefit from different options at different points in his/her recovery. This may seem to be a self-evident assertion; however, focus on this issue is needed since NA currently has a near monopoly in the “recovery market”. The promotion of other groups was hampered by the fact that NA participants only spoke about their own fellowship, and did not recommend others. Our present respondents ascribed this behavior to arrogance or the devaluation of other available resources. From the perspective of NA members, this omission may follow the NA traditions that state that NA participants should not convey opinions on “outside” issues (tradition 10). However, NA participants are also not supposed to be zealous missionaries for their fellowship—their relation with the outside world (other possible attendees and public relations) should rely on attraction rather than promotion (tradition 11). Thus, it would be in accordance with the humility principle in the 12 traditions that NA participants acknowledge that NA may not be the only solution to addiction.

### Frustration with NA

The frustration described by many of the respondents should not be overlooked. NA was highly important to the respondents during critical turning points of their lives. However, this important role of NA was contrasted by the tension and frustration that led to their desistance from NA. Leaving NA was even described as feeling like apostasy, underlining the dilemma that leaving a “tight” social fellowship can arouse feelings of stigmatization that may potentially depress self-esteem [26]. On a deeper level, the respondents’ frustration can also be thought of as “identity ambivalence” ([27], p. 131). The participants might have been afraid to weaken their identity as “addicts”, because the NA literature suggests that this self-reminding is a crucial defense mechanism against relapse ([16], p. 12). As the respondents finally left the fellowship because they were unable to integrate their social identity in NA with a new social identity (e.g., being an “ordinary” citizen), some of them greatly appreciated the opportunity to talk about this separation dilemma and the associated frustration. We interpreted that the respondents seemed to benefit from the interviews as a kind of debriefing from frustrated experiences. Mutual help is mainly discussed in beneficial terms, and it is vital for professionals in the addiction field to motivate and encourage participation [1, 5]. However, it is also important to be aware of potential problems related to participation, to enable their prevention and mitigation. Examples include the respondents’ negative reactions to the informal hierarchy and the “conformity pressure”. Notably, a certain conformity pressure is actually considered to be a mechanism behind the transformative power in SHGs, and should not automatically be regarded as a negative factor. The programs and narratives in mutual help contexts can provide a normative structure that functions as an agent for identity transformation and change—shaping members’ values and self-understanding [28, 29]. However, the present respondents perceived that this normative power also was used to support some members’ standings in the fellowship, and their frustration may have partly been brought about by the actions of seasoned members who tried to bolster their position rather than live up to the NA ideal of a leader who serves.

Another reason for disengagement was disappointment with how concurrent problems (such as mental health problems or recurrent trauma) were met by persons within NA. Prior research has shown that patients with a comorbid mental disorder can benefit from TSG participation [30], but that problem severity may influence affiliation and benefits [31]. Our present findings speak to the introspective process, whereby persons with a comorbidity might devaluate themselves because they were unable to live up to the informal standards of “success” in the group. Our respondents pointed out that helpful alternatives include NGOs involving activities other than just “talk groups”, and groups with greater focus on dialogue and supervision.

Similar to our present findings, previous case stories describe negative experiences of trauma survivors in 12-step groups [32]. Although some authors advocate for how the 12 steps can be used in a trauma-informed and trauma-friendly way [32, 33], whether this actually occurs depends on sponsorship and a sound interpretation of the 12-step language. In line with the original purpose of the 12 steps—which were designed to address addiction only, not trauma or other mental health issues—the 12 steps do not focus on harm done *to* the participant but only on the harm the participant has done to him/herself and others [34]. Thus, it would be an uninformed approach to step 5 to term trauma survivors’ resentment and anger as “defects of character” and to thereby risk discrediting members’ understandable and well-founded feelings of resentment and anger during the process of obtaining healing. The lifetime prevalence of physical and sexual abuse is high among patients in treatment for a SUD, with a large study reporting past victimization among roughly one-third of women and one-tenth of men [35]. Thus, such victimization is likely quite common among persons coming to NA. When a sponsor lacks personal experience in this area or is not aware of the extra challenges faced by trauma survivors, there is a risk that the burden of blame and guilt will be increased through step 5. In accordance with the self-help ethos that self-help is based on experiential knowledge [36], it would be wise for sponsors to be humble in areas where they lack personal experience and to restrain an immediate inclination to act as therapists.

### Measures to improve participants’ benefits of participation

A somewhat hidden message in our findings was that the respondents wished that there had been more information available on how to use TSGs. To our knowledge, some NA groups have occasional theme meetings about the sponsor–sponsee relationship and, for example, will emphasize that you can choose another sponsor if you are dissatisfied with something about your present partnership. Members should also be helped to understand what ought not be shared in meetings, as there is a risk of being overly open due to feeling “blinded” by initial enthusiasm. This may also be a task for the addiction professionals who recommend these fellowships. Attendees should take some time to adjust to the group environment, and should be skeptical about sharing their innermost secrets in meetings, at least early on. Likewise, professionals could recommend that potential participants with concurrent problems (e.g., trauma experiences) should also seek out relevant fellowships to achieve experiential support for that problem—for example, a support group for sexual abuse or childhood trauma. After all, mutual help is about sharing experiences, and a person might have more than one problem to cope with.

### Methodological considerations and implications

Due to its explorative nature, this study cannot cover the full range of the phenomenon, but rather highlight selected patterns relevant to the study aim [37]. The relatively low number of participants was justified based on the purposive sampling procedure that resulted in a sample with experiences highly specific for the study objectives. Thus, we believe that the study had sufficient information power [37]. We note that our findings may not be directly transferable to other TSGs or to experiences with NA in other countries. As has been noted by others, the milieu of a local TSG can vary considerably across locations, depending on the local culture and how the 12 steps have been implemented in the group [38]. However, our in-depth findings are similar to qualitative comments from a prior study in the TSGs’ country of origin [25]; therefore, we believe that our findings may be valuable in treatment and community settings outside Norway.

Few of our respondents left NA within their first year. Thus, additional studies are needed to examine early attrition from NA [7]. As some of the authors have worked in a 12-step-friendly treatment environment, we acknowledge that we have previously had the inclination to dismiss negative information about TSGs. In the present study, we intentionally set out to challenge our previous assumptions, and the interviews and analyses contributed to broadening our perspectives in this area. We note that the study was part of a larger qualitative study about NA and a previous paper examined views of seasoned, present-day members [39].

An overall implication of this study for addiction professionals is to avoid contributing to an unseemly favoritism of certain groups, by conveying that it may be equally beneficial to seek out alternatives. As Hänninen and Koski-Jännes have noted, there are many routes to recovery and “Clients ….should be encouraged to create and express a story that fits their own experience, to make full use of the cultural stock of stories and not to comply blindly with any pre-existing narrative model” ([40], p. 1847).

## Conclusions

The results of the present study highlighted some potential problems related to TSG participation in the setting of Norway, and adds regional texture to the largely Anglo-American literature. Recognition of these problems can enable the prevention of negative experiences and possible harms to individuals, and elucidates the need to have various support alternatives available for persons with SUDs.

## Data Availability

Not applicable (the transcripts are in Norwegian).

## Abbreviations

AA: Alcoholics Anonymous
NA: Narcotics Anonymous
NGO: Non-governmental organizations
OMT: Opioid maintenance treatment
PAL: Peer ALternatives for Addiction study
SHG: Self-help groups
SUD: Substance use disorders
TSG: 12-step groups
WFS: Women for sobriety
